# 9-(3,4-Dimeth­oxy­phen­yl)-3,3,6,6-tetra­methyl-4,5,6,9-tetra­hydro-3*H*-xanthene-1,8(2*H*,7*H*)-dione

**DOI:** 10.1107/S1600536811023014

**Published:** 2011-06-18

**Authors:** Sayed Hasan Mehdi, Othman Sulaiman, Raza Murad Ghalib, Chin Sing Yeap, Hoong-Kun Fun

**Affiliations:** a School of Industrial Technology, Universiti Sains Malaysia, 11800 USM, Penang, Malaysia; b X-ray Crystallography Unit, School of Physics, Universiti Sains Malaysia, 11800 USM, Penang, Malaysia

## Abstract

The asymmetric unit of the title xanthene compound, C_25_H_30_O_5_, contains two mol­ecules in which the pyran ring and the dimeth­oxy­phenyl ring are nearly perpendicular to one another [dihedral angles = 86.81 (8) and 84.45 (9)°]. One of the meth­oxy groups in one mol­ecule is twisted away from the phenyl ring [C—O—C—C torsion angle = −103.40 (16)°]. The pyran ring adopts a boat conformation whereas the two fused cyclo­hexane rings adopt envelope conformations in both mol­ecules. In the crystal, mol­ecules are linked into a three-dimensional network by C—H⋯O hydrogen bonds.

## Related literature

For applications of xanthene derivatives, see: Lambert *et al.* (1997 ▶); Hideo (1981 ▶); Poupelin *et al.* (1978 ▶); Menchen *et al.* (2003 ▶); Banerjee & Mukherjee (1981 ▶); Ravindranath & Seshadri (1973) ▶. For the synthesis of xanthene and 1,8-dioxoocta­hydroxanthene derivatives with or without the use of a catalyst, see: Fan *et al.* (2005 ▶); Jin *et al.* (2005 ▶); Srihari *et al.* (2008 ▶). For a related structure, see: Mehdi *et al.* (2011 ▶). For the stability of the temperature controller used in the data collection, see: Cosier & Glazer (1986 ▶). For ring conformations, see: Cremer & Pople (1975 ▶).
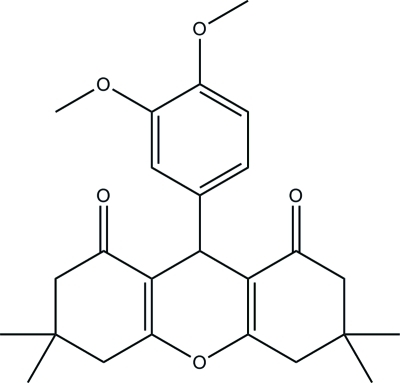


## Experimental

### 

#### Crystal data

C_25_H_30_O_5_
*M* *_r_* = 410.49
Triclinic, 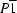

*a* = 9.4895 (7) Å
*b* = 10.2283 (7) Å
*c* = 23.3218 (16) Å
α = 85.872 (4)°
β = 86.537 (4)°
γ = 74.425 (3)°
*V* = 2172.9 (3) Å^3^
*Z* = 4
Mo *K*α radiation
μ = 0.09 mm^−1^
*T* = 100 K
0.42 × 0.39 × 0.20 mm

#### Data collection

Bruker SMART APEXII CCD diffractometer
Absorption correction: multi-scan (*SADABS*; Bruker, 2009 ▶) *T*_min_ = 0.965, *T*_max_ = 0.983
44431 measured reflections
11497 independent reflections
8871 reflections with *I* > 2σ(*I*)
*R*_int_ = 0.040

#### Refinement

*R*[*F* ^2^ > 2σ(*F* ^2^)] = 0.054
*wR*(*F* ^2^) = 0.127
*S* = 1.01
11497 reflections
553 parameters
H-atom parameters constrained
Δρ_max_ = 0.42 e Å^−3^
Δρ_min_ = −0.28 e Å^−3^

### 

Data collection: *APEX2* (Bruker, 2009 ▶); cell refinement: *SAINT* (Bruker, 2009 ▶); data reduction: *SAINT*; program(s) used to solve structure: *SHELXTL* (Sheldrick, 2008 ▶); program(s) used to refine structure: *SHELXTL*; molecular graphics: *SHELXTL*; software used to prepare material for publication: *SHELXTL* and *PLATON* (Spek, 2009 ▶).

## Supplementary Material

Crystal structure: contains datablock(s) global, I. DOI: 10.1107/S1600536811023014/hb5885sup1.cif

Structure factors: contains datablock(s) I. DOI: 10.1107/S1600536811023014/hb5885Isup2.hkl

Supplementary material file. DOI: 10.1107/S1600536811023014/hb5885Isup3.cml

Additional supplementary materials:  crystallographic information; 3D view; checkCIF report

## Figures and Tables

**Table 1:** Hydrogen-bond geometry (Å, °)

*D*—H⋯*A*	*D*—H	H⋯*A*	*D*⋯*A*	*D*—H⋯*A*
C10*A*—H10*A*⋯O4*A*^i^	0.99	2.38	3.2817 (19)	152
C18*A*—H18*A*⋯O2*A*^ii^	0.95	2.35	3.2943 (19)	176
C18*B*—H18*B*⋯O2*B*^iii^	0.95	2.45	3.4000 (19)	175
C20*A*—H20*A*⋯O3*A*^iv^	0.98	2.59	3.4144 (19)	142
C24*B*—H24*D*⋯O4*A*^v^	0.98	2.48	3.453 (2)	171

Symmetry codes: (i) 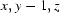; (ii) 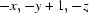; (iii) 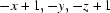; (iv) 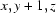; (v) 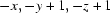.

**Fig. 1.**
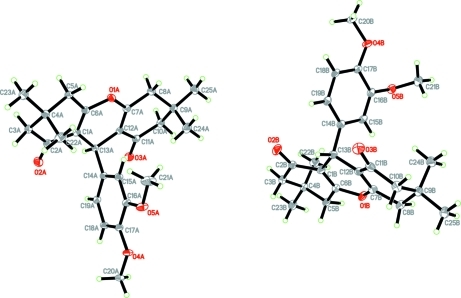
The molecular structure of (I) with 50% probability ellipsoids for non-H atoms.

**Fig. 2.**
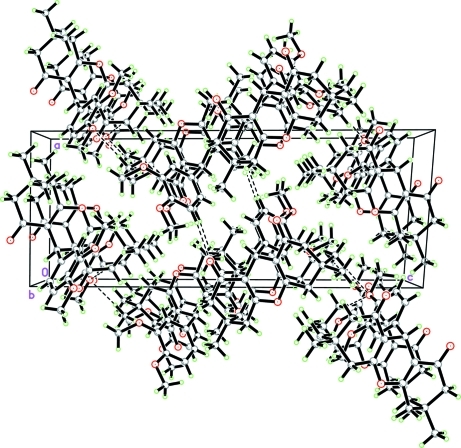
The packing of (I), viewed down b axis, showing molecules linked into a three-dimensional network. Hydrogen bonds are shown as dashed lines.

## supplementary crystallographic information

### Comment

Synthesis of xanthene derivatives is currently of great interest and has attracted considerable attention by chemists because of their biological and pharmaceutical properties as antiviral (Lambert *et al.*, 1997), antibacterial (Hideo, 1981), and anti-inflammatory (Poupelin *et al.*, 1978). Xanthenes derivatives also find use as dyes, fluorescent material for visualization of biomolecules and in laser technologies (Menchen *et al.*, 2003; Banerjee & Mukherjee, 1981). Several natural occurring polycyclic compounds containing xanthene nucleus are also reported (Ravindranath & Seshadri, 1973). Synthesis of xanthene and 1,8-dioxooctahydroxanthene derivatives have been reported in literature under the different reaction conditions with or without the use of catalyst (Fan *et al.*, 2005; Jin *et al.*, 2005; Srihari *et al.*, 2008). Here we are reporting the synthesis of title compound, (I). The structure of the title compound was established on the basis of its IR, ^1^H NMR, ^13^C NMR spectra and finally confirmed by X-ray analysis.

The asymmetric unit of (I) consists of two crystallographically independent molecules, *A* and *B* (Fig. 1). The geometric parameters and the conformations are very similar to the previously reported structure (Mehdi *et al.*, 2011). However, only the dimethoxyphenyl of molecule *B* is different from others. The dimethoxyphenyl grouping in molecule *A* is almost planar [C20A–O4A–C17A–C18A = -1.9 (2)° and C21A–O5A–C16A–C17A = 4.6 (2)°] whereas for molecule *B* is not planar [C20B–O4B–C17B–C18B = -6.1 (2)° and C21B–O5B–C16B–C15B = -103.40 (16)°]. Similarly, the mean plane of pyran ring and the dimethoxyphenyl ring for both *A* and *B* molecules are nearly perpendicular to one another with the dihedral angles between them being 86.81 (8) and 84.45 (9)°, respectively. For both molecules, the two cyclohexane rings adopt envelope conformations whereas the pyran ring adopts a boat conformation (Cremer & Pople, 1975).

In the crystal, the molecules are linked into a three-dimensional network (Fig. 2) by C—H···O hydrogen bonds (Table 1).

### Experimental

A mixture of dimedone (1.40 g m, 10 mmol) and veratraldehyde (1.66 g m, 10 mmol) was heated in 25 ml of glacial acetic acid for three hours. Completion of the reaction was monitored by TLC. The reaction mixture was dried on rotary evaporator under reduced pressure. The crude mixture thus obtained was successively treated with di ethyl ether chloroform and ethanol. The ethanol fraction on crystallization furnished yellow blocks of (I) (m pt. 208 °C, Yield 90%). IR (KBr) ν_max_: 3085, 3005, 2956, 2929, 2870, 2822, 1667, 1624, 1515, 1467, 1419, 1358, 1227, 1141, 1105, 1023, 851, 830, 751 cm^-1. 1^H NMR (300 MHz, DMSO-d_6_): δ 6.75–6.92 (3*H*, m, Aromatic protons), 4.72 (1*H*, s), 3.83 (3*H*, s), 3.78 (3*H*, s), 2.40 (4*H*, s), 2.17 (4*H*, s), 1.05 (6*H*, s), 1.02 (6*H*, s). ^13^C NMR (75 MHz, DMSO-d_6_): δ 27.2, 36.8, 42.4, 52.2, 56.4, 112.2, 114.6, 115.2, 123.4, 136.8, 146.6, 148.4, 198.2. IR spectrum was taken on Shimadzu IR-408 Perkin Elmer 1800 (FTIR). ^1^H NMR was recorded on Bruker Avance 300 MHz with TMS as an internal standard and 75 MHz for ^13^C NMR. Spectrum was recorded in DMSO-d_6_. The melting point was taken on Thermo Fisher digital melting point apparatus of IA9000 series and is uncorrected.

### Refinement

All hydrogen atoms were positioned geomatrically [C–H = 0.95–1.00 Å] and refined using a riding model, with *U*_iso_(H) = 1.2 or 1.5 *U*_eq_(C). A rotating-group model were applied for methyl groups.

### Crystal data

**Table:**

C_25_H_30_O_5_	*Z* = 4
*M**_r_* = 410.49	*F*(000) = 880
Triclinic, *P*1	*D*_x_ = 1.255 Mg m^−^^3^
Hall symbol: -P 1	Mo *K*α radiation, λ = 0.71073 Å
*a* = 9.4895 (7) Å	Cell parameters from 9984 reflections
*b* = 10.2283 (7) Å	θ = 2.2–30.0°
*c* = 23.3218 (16) Å	µ = 0.09 mm^−^^1^
α = 85.872 (4)°	*T* = 100 K
β = 86.537 (4)°	Block, yellow
γ = 74.425 (3)°	0.42 × 0.39 × 0.20 mm
*V* = 2172.9 (3) Å^3^	

### Data collection

**Table:**

Bruker SMART APEXII CCD diffractometer	11497 independent reflections
Radiation source: fine-focus sealed tube	8871 reflections with *I* > 2σ(*I*)
graphite	*R*_int_ = 0.040
φ and ω scans	θ_max_ = 29.0°, θ_min_ = 0.9°
Absorption correction: multi-scan (*SADABS*; Bruker, 2009)	*h* = −12→12
*T*_min_ = 0.965, *T*_max_ = 0.983	*k* = −13→13
44431 measured reflections	*l* = −31→31

### Refinement

**Table:**

Refinement on *F*^2^	Primary atom site location: structure-invariant direct methods
Least-squares matrix: full	Secondary atom site location: difference Fourier map
*R*[*F*^2^ > 2σ(*F*^2^)] = 0.054	Hydrogen site location: inferred from neighbouring sites
*wR*(*F*^2^) = 0.127	H-atom parameters constrained
*S* = 1.01	*w* = 1/[σ^2^(*F*_o_^2^) + (0.0552*P*)^2^ + 1.1161*P*] where *P* = (*F*_o_^2^ + 2*F*_c_^2^)/3
11497 reflections	(Δ/σ)_max_ = 0.001
553 parameters	Δρ_max_ = 0.42 e Å^−^^3^
0 restraints	Δρ_min_ = −0.28 e Å^−^^3^

### Special details

**Table:**

Experimental. The crystal was placed in the cold stream of an Oxford Cryosystems Cobra open-flow nitrogen cryostat (Cosier & Glazer, 1986) operating at 100.0 (1) K.
Geometry. All e.s.d.'s (except the e.s.d. in the dihedral angle between two l.s. planes) are estimated using the full covariance matrix. The cell e.s.d.'s are taken into account individually in the estimation of e.s.d.'s in distances, angles and torsion angles; correlations between e.s.d.'s in cell parameters are only used when they are defined by crystal symmetry. An approximate (isotropic) treatment of cell e.s.d.'s is used for estimating e.s.d.'s involving l.s. planes.
Refinement. Refinement of *F*^2^ against ALL reflections. The weighted *R*-factor *wR* and goodness of fit *S* are based on *F*^2^, conventional *R*-factors *R* are based on *F*, with *F* set to zero for negative *F*^2^. The threshold expression of *F*^2^ > σ(*F*^2^) is used only for calculating *R*-factors(gt) *etc*. and is not relevant to the choice of reflections for refinement. *R*-factors based on *F*^2^ are statistically about twice as large as those based on *F*, and *R*- factors based on ALL data will be even larger.

### Fractional atomic coordinates and isotropic or equivalent isotropic displacement parameters (Å^2^)

**Table:**

	*x*	*y*	*z*	*U*_iso_*/*U*_eq_	
O1A	0.51121 (11)	0.01893 (10)	0.13242 (4)	0.0167 (2)	
O2A	0.30935 (12)	0.27866 (12)	−0.03360 (5)	0.0222 (2)	
O3A	−0.00427 (11)	0.10581 (11)	0.13190 (5)	0.0194 (2)	
O4A	0.03071 (11)	0.72025 (10)	0.14408 (5)	0.0170 (2)	
O5A	0.26685 (12)	0.56744 (11)	0.18780 (5)	0.0212 (2)	
C1A	0.39692 (15)	0.15892 (14)	0.05242 (6)	0.0130 (3)	
C2A	0.41570 (16)	0.22544 (14)	−0.00480 (6)	0.0146 (3)	
C3A	0.56930 (16)	0.22129 (15)	−0.02765 (6)	0.0163 (3)	
H3AA	0.5662	0.3059	−0.0516	0.020*	
H3AB	0.6034	0.1442	−0.0529	0.020*	
C4A	0.68098 (16)	0.20643 (15)	0.01876 (6)	0.0156 (3)	
C5A	0.67052 (16)	0.08554 (15)	0.06052 (6)	0.0153 (3)	
H5AA	0.7132	−0.0002	0.0412	0.018*	
H5AB	0.7289	0.0847	0.0944	0.018*	
C6A	0.51680 (16)	0.09159 (14)	0.08028 (6)	0.0137 (3)	
C7A	0.37572 (16)	0.00791 (14)	0.15400 (6)	0.0147 (3)	
C8A	0.38861 (16)	−0.08773 (15)	0.20637 (6)	0.0176 (3)	
H8AA	0.4737	−0.0831	0.2280	0.021*	
H8AB	0.4064	−0.1818	0.1943	0.021*	
C9A	0.24950 (16)	−0.05339 (15)	0.24603 (6)	0.0167 (3)	
C10A	0.11824 (17)	−0.04029 (15)	0.20912 (6)	0.0173 (3)	
H10A	0.1195	−0.1320	0.1977	0.021*	
H10B	0.0276	−0.0067	0.2330	0.021*	
C11A	0.11168 (16)	0.05299 (14)	0.15528 (6)	0.0150 (3)	
C12A	0.25000 (16)	0.07503 (14)	0.12990 (6)	0.0133 (3)	
C13A	0.24385 (15)	0.17523 (14)	0.07831 (6)	0.0129 (3)	
H13A	0.1811	0.1549	0.0490	0.015*	
C14A	0.18013 (15)	0.32153 (14)	0.09553 (6)	0.0132 (3)	
C15A	0.25380 (16)	0.37360 (14)	0.13478 (6)	0.0151 (3)	
H15A	0.3402	0.3170	0.1511	0.018*	
C16A	0.20193 (16)	0.50682 (15)	0.15011 (6)	0.0150 (3)	
C17A	0.07316 (15)	0.59013 (14)	0.12625 (6)	0.0140 (3)	
C18A	−0.00021 (16)	0.53859 (14)	0.08766 (6)	0.0151 (3)	
H18A	−0.0873	0.5945	0.0716	0.018*	
C19A	0.05405 (15)	0.40429 (14)	0.07237 (6)	0.0143 (3)	
H19A	0.0037	0.3694	0.0457	0.017*	
C20A	−0.09712 (16)	0.80929 (15)	0.11921 (7)	0.0169 (3)	
H20A	−0.1143	0.9000	0.1337	0.025*	
H20B	−0.1820	0.7738	0.1296	0.025*	
H20C	−0.0825	0.8150	0.0772	0.025*	
C21A	0.4031 (2)	0.48823 (19)	0.20905 (9)	0.0326 (4)	
H21A	0.4398	0.5414	0.2351	0.049*	
H21B	0.4740	0.4631	0.1767	0.049*	
H21C	0.3891	0.4057	0.2300	0.049*	
C22A	0.65038 (18)	0.33737 (16)	0.05122 (7)	0.0223 (3)	
H22A	0.6608	0.4127	0.0244	0.033*	
H22B	0.7203	0.3249	0.0817	0.033*	
H22C	0.5505	0.3580	0.0683	0.033*	
C23A	0.83548 (17)	0.17712 (16)	−0.00895 (7)	0.0203 (3)	
H23A	0.8439	0.2542	−0.0352	0.031*	
H23B	0.8548	0.0949	−0.0305	0.031*	
H23C	0.9068	0.1634	0.0211	0.031*	
C24A	0.23732 (19)	0.07937 (17)	0.27513 (7)	0.0244 (3)	
H24A	0.1489	0.1000	0.3004	0.037*	
H24B	0.2317	0.1537	0.2457	0.037*	
H24C	0.3236	0.0692	0.2979	0.037*	
C25A	0.25761 (18)	−0.16875 (17)	0.29255 (7)	0.0235 (3)	
H25A	0.1710	−0.1456	0.3188	0.035*	
H25B	0.3460	−0.1813	0.3142	0.035*	
H25C	0.2610	−0.2531	0.2744	0.035*	
O1B	−0.06753 (11)	0.41491 (11)	0.63022 (4)	0.0175 (2)	
O2B	0.17623 (12)	0.22238 (12)	0.46132 (5)	0.0258 (3)	
O3B	0.43753 (12)	0.39314 (12)	0.61832 (5)	0.0246 (3)	
O4B	0.48890 (11)	−0.23710 (10)	0.66652 (5)	0.0195 (2)	
O5B	0.23066 (11)	−0.09300 (11)	0.71319 (4)	0.0182 (2)	
C1B	0.06703 (16)	0.30810 (15)	0.54858 (6)	0.0156 (3)	
C2B	0.06302 (17)	0.26311 (15)	0.49034 (6)	0.0177 (3)	
C3B	−0.08492 (17)	0.27615 (16)	0.46638 (6)	0.0195 (3)	
H3BA	−0.0750	0.2027	0.4398	0.023*	
H3BB	−0.1144	0.3638	0.4436	0.023*	
C4B	−0.20776 (16)	0.26930 (15)	0.51161 (6)	0.0167 (3)	
C5B	−0.20940 (16)	0.37152 (15)	0.55704 (6)	0.0173 (3)	
H5BA	−0.2512	0.4648	0.5405	0.021*	
H5BB	−0.2736	0.3555	0.5904	0.021*	
C6B	−0.06074 (16)	0.36086 (15)	0.57727 (6)	0.0157 (3)	
C7B	0.05787 (16)	0.44058 (15)	0.64789 (6)	0.0160 (3)	
C8B	0.02695 (17)	0.52491 (16)	0.69919 (7)	0.0187 (3)	
H8BA	−0.0426	0.4924	0.7262	0.022*	
H8BB	−0.0201	0.6205	0.6867	0.022*	
C9B	0.16583 (17)	0.51902 (15)	0.73048 (6)	0.0180 (3)	
C10B	0.28224 (17)	0.54196 (16)	0.68493 (7)	0.0195 (3)	
H10C	0.2476	0.6339	0.6659	0.023*	
H10D	0.3736	0.5379	0.7042	0.023*	
C11B	0.31460 (16)	0.43762 (16)	0.63990 (7)	0.0185 (3)	
C12B	0.18952 (16)	0.39419 (14)	0.62110 (6)	0.0158 (3)	
C13B	0.21341 (16)	0.29660 (15)	0.57359 (6)	0.0156 (3)	
H13B	0.2789	0.3248	0.5427	0.019*	
C14B	0.28762 (16)	0.15174 (15)	0.59614 (6)	0.0153 (3)	
C15B	0.22690 (16)	0.09184 (15)	0.64401 (6)	0.0165 (3)	
H15B	0.1366	0.1405	0.6613	0.020*	
C16B	0.29577 (16)	−0.03643 (15)	0.66644 (6)	0.0154 (3)	
C17B	0.42943 (16)	−0.11082 (15)	0.64140 (6)	0.0150 (3)	
C18B	0.49040 (16)	−0.05217 (15)	0.59372 (6)	0.0163 (3)	
H18B	0.5805	−0.1007	0.5763	0.020*	
C19B	0.41944 (16)	0.07738 (15)	0.57161 (6)	0.0164 (3)	
H19B	0.4621	0.1159	0.5390	0.020*	
C20B	0.61663 (17)	−0.31935 (16)	0.63789 (7)	0.0215 (3)	
H20D	0.6444	−0.4098	0.6577	0.032*	
H20E	0.6974	−0.2766	0.6385	0.032*	
H20F	0.5954	−0.3278	0.5979	0.032*	
C21B	0.29838 (18)	−0.08858 (17)	0.76608 (7)	0.0221 (3)	
H21D	0.2459	−0.1256	0.7979	0.033*	
H21E	0.2950	0.0058	0.7727	0.033*	
H21F	0.4006	−0.1429	0.7638	0.033*	
C22B	−0.18128 (19)	0.12487 (16)	0.53976 (7)	0.0226 (3)	
H22D	−0.1820	0.0612	0.5104	0.034*	
H22E	−0.2588	0.1223	0.5692	0.034*	
H22F	−0.0861	0.0989	0.5576	0.034*	
C23B	−0.35524 (18)	0.30927 (17)	0.48314 (7)	0.0227 (3)	
H23D	−0.3562	0.2436	0.4547	0.034*	
H23E	−0.3704	0.4002	0.4640	0.034*	
H23G	−0.4338	0.3098	0.5125	0.034*	
C24B	0.22219 (19)	0.37987 (17)	0.76248 (7)	0.0246 (4)	
H24D	0.1452	0.3623	0.7895	0.037*	
H24G	0.3083	0.3795	0.7838	0.037*	
H24E	0.2491	0.3089	0.7347	0.037*	
C25B	0.13179 (19)	0.63007 (17)	0.77360 (7)	0.0226 (3)	
H25D	0.0522	0.6180	0.8005	0.034*	
H25E	0.1020	0.7194	0.7530	0.034*	
H25F	0.2193	0.6241	0.7950	0.034*	

### Atomic displacement parameters (Å^2^)

**Table:**

	*U*^11^	*U*^22^	*U*^33^	*U*^12^	*U*^13^	*U*^23^
O1A	0.0117 (5)	0.0192 (5)	0.0173 (5)	−0.0019 (4)	−0.0001 (4)	0.0038 (4)
O2A	0.0161 (5)	0.0282 (6)	0.0198 (5)	−0.0020 (4)	−0.0034 (4)	0.0031 (5)
O3A	0.0142 (5)	0.0211 (5)	0.0228 (5)	−0.0046 (4)	−0.0010 (4)	−0.0011 (4)
O4A	0.0151 (5)	0.0109 (5)	0.0230 (5)	0.0012 (4)	−0.0043 (4)	−0.0027 (4)
O5A	0.0169 (5)	0.0177 (5)	0.0284 (6)	0.0006 (4)	−0.0105 (5)	−0.0084 (5)
C1A	0.0122 (7)	0.0116 (6)	0.0153 (7)	−0.0027 (5)	0.0003 (5)	−0.0035 (5)
C2A	0.0158 (7)	0.0120 (6)	0.0157 (7)	−0.0026 (5)	−0.0010 (6)	−0.0036 (5)
C3A	0.0155 (7)	0.0180 (7)	0.0140 (7)	−0.0027 (5)	0.0004 (5)	0.0003 (6)
C4A	0.0137 (7)	0.0157 (7)	0.0168 (7)	−0.0035 (5)	0.0008 (5)	−0.0004 (5)
C5A	0.0125 (7)	0.0148 (7)	0.0166 (7)	−0.0005 (5)	−0.0002 (5)	−0.0002 (5)
C6A	0.0147 (7)	0.0117 (6)	0.0145 (6)	−0.0029 (5)	0.0002 (5)	−0.0016 (5)
C7A	0.0140 (7)	0.0136 (7)	0.0166 (7)	−0.0036 (5)	0.0015 (5)	−0.0026 (5)
C8A	0.0159 (7)	0.0159 (7)	0.0186 (7)	−0.0013 (5)	−0.0004 (6)	0.0021 (6)
C9A	0.0178 (7)	0.0170 (7)	0.0150 (7)	−0.0047 (6)	0.0019 (6)	−0.0015 (6)
C10A	0.0183 (7)	0.0172 (7)	0.0176 (7)	−0.0072 (6)	0.0021 (6)	−0.0021 (6)
C11A	0.0156 (7)	0.0138 (7)	0.0164 (7)	−0.0042 (5)	−0.0002 (6)	−0.0051 (5)
C12A	0.0144 (7)	0.0107 (6)	0.0148 (6)	−0.0028 (5)	0.0005 (5)	−0.0031 (5)
C13A	0.0111 (6)	0.0119 (6)	0.0148 (6)	−0.0011 (5)	−0.0009 (5)	−0.0019 (5)
C14A	0.0117 (7)	0.0132 (6)	0.0140 (6)	−0.0023 (5)	0.0014 (5)	−0.0005 (5)
C15A	0.0118 (7)	0.0146 (7)	0.0173 (7)	−0.0005 (5)	−0.0022 (5)	−0.0005 (5)
C16A	0.0134 (7)	0.0155 (7)	0.0159 (7)	−0.0031 (5)	−0.0015 (5)	−0.0022 (5)
C17A	0.0125 (7)	0.0121 (6)	0.0162 (7)	−0.0017 (5)	0.0009 (5)	0.0000 (5)
C18A	0.0121 (7)	0.0139 (7)	0.0174 (7)	−0.0003 (5)	−0.0015 (5)	0.0007 (5)
C19A	0.0123 (7)	0.0155 (7)	0.0153 (7)	−0.0036 (5)	−0.0014 (5)	−0.0023 (5)
C20A	0.0130 (7)	0.0129 (7)	0.0219 (7)	0.0016 (5)	−0.0030 (6)	−0.0001 (6)
C21A	0.0249 (9)	0.0268 (9)	0.0440 (11)	0.0035 (7)	−0.0212 (8)	−0.0127 (8)
C22A	0.0210 (8)	0.0189 (8)	0.0279 (8)	−0.0064 (6)	0.0011 (7)	−0.0056 (6)
C23A	0.0152 (7)	0.0239 (8)	0.0215 (8)	−0.0056 (6)	0.0026 (6)	0.0004 (6)
C24A	0.0281 (9)	0.0249 (8)	0.0209 (8)	−0.0067 (7)	−0.0017 (7)	−0.0075 (6)
C25A	0.0228 (8)	0.0261 (8)	0.0203 (8)	−0.0062 (6)	0.0020 (6)	0.0033 (6)
O1B	0.0123 (5)	0.0217 (5)	0.0192 (5)	−0.0047 (4)	0.0028 (4)	−0.0084 (4)
O2B	0.0199 (6)	0.0333 (7)	0.0213 (6)	−0.0025 (5)	0.0077 (5)	−0.0068 (5)
O3B	0.0141 (5)	0.0300 (6)	0.0296 (6)	−0.0065 (5)	0.0039 (5)	−0.0022 (5)
O4B	0.0171 (5)	0.0149 (5)	0.0229 (5)	0.0007 (4)	0.0059 (4)	−0.0018 (4)
O5B	0.0169 (5)	0.0196 (5)	0.0184 (5)	−0.0065 (4)	0.0052 (4)	−0.0013 (4)
C1B	0.0147 (7)	0.0142 (7)	0.0171 (7)	−0.0026 (5)	0.0014 (6)	−0.0010 (5)
C2B	0.0197 (7)	0.0156 (7)	0.0163 (7)	−0.0032 (6)	0.0036 (6)	−0.0003 (6)
C3B	0.0215 (8)	0.0205 (7)	0.0152 (7)	−0.0035 (6)	0.0005 (6)	−0.0016 (6)
C4B	0.0169 (7)	0.0176 (7)	0.0161 (7)	−0.0050 (6)	0.0010 (6)	−0.0039 (6)
C5B	0.0141 (7)	0.0180 (7)	0.0190 (7)	−0.0027 (5)	0.0015 (6)	−0.0046 (6)
C6B	0.0158 (7)	0.0160 (7)	0.0154 (7)	−0.0042 (5)	0.0003 (6)	−0.0024 (5)
C7B	0.0139 (7)	0.0152 (7)	0.0192 (7)	−0.0042 (5)	0.0004 (6)	−0.0016 (6)
C8B	0.0164 (7)	0.0185 (7)	0.0213 (7)	−0.0043 (6)	0.0018 (6)	−0.0058 (6)
C9B	0.0183 (7)	0.0189 (7)	0.0174 (7)	−0.0062 (6)	0.0015 (6)	−0.0019 (6)
C10B	0.0177 (7)	0.0223 (8)	0.0212 (7)	−0.0103 (6)	−0.0015 (6)	0.0003 (6)
C11B	0.0157 (7)	0.0199 (7)	0.0195 (7)	−0.0054 (6)	0.0000 (6)	0.0044 (6)
C12B	0.0157 (7)	0.0138 (7)	0.0169 (7)	−0.0027 (5)	0.0004 (6)	0.0005 (5)
C13B	0.0113 (7)	0.0169 (7)	0.0176 (7)	−0.0028 (5)	0.0043 (5)	−0.0011 (6)
C14B	0.0122 (7)	0.0164 (7)	0.0177 (7)	−0.0037 (5)	0.0007 (5)	−0.0041 (6)
C15B	0.0113 (7)	0.0184 (7)	0.0191 (7)	−0.0030 (5)	0.0047 (6)	−0.0047 (6)
C16B	0.0134 (7)	0.0179 (7)	0.0161 (7)	−0.0066 (5)	0.0034 (5)	−0.0034 (6)
C17B	0.0117 (7)	0.0162 (7)	0.0178 (7)	−0.0040 (5)	−0.0005 (5)	−0.0047 (6)
C18B	0.0116 (7)	0.0194 (7)	0.0174 (7)	−0.0028 (5)	0.0037 (5)	−0.0068 (6)
C19B	0.0143 (7)	0.0191 (7)	0.0156 (7)	−0.0046 (6)	0.0035 (6)	−0.0030 (6)
C20B	0.0174 (8)	0.0180 (7)	0.0254 (8)	0.0016 (6)	0.0036 (6)	−0.0052 (6)
C21B	0.0216 (8)	0.0251 (8)	0.0196 (7)	−0.0067 (6)	0.0037 (6)	−0.0024 (6)
C22B	0.0246 (8)	0.0186 (8)	0.0256 (8)	−0.0075 (6)	−0.0003 (7)	−0.0016 (6)
C23B	0.0199 (8)	0.0283 (9)	0.0210 (8)	−0.0070 (6)	−0.0019 (6)	−0.0054 (7)
C24B	0.0256 (9)	0.0243 (8)	0.0211 (8)	−0.0036 (7)	0.0016 (7)	0.0028 (6)
C25B	0.0243 (8)	0.0249 (8)	0.0211 (8)	−0.0100 (7)	−0.0022 (6)	−0.0045 (6)

### Geometric parameters (Å, °)

**Table:**

O1A—C7A	1.3822 (17)	O1B—C7B	1.3774 (18)
O1A—C6A	1.3847 (16)	O1B—C6B	1.3800 (18)
O2A—C2A	1.2248 (18)	O2B—C2B	1.2240 (18)
O3A—C11A	1.2291 (18)	O3B—C11B	1.2240 (18)
O4A—C17A	1.3704 (17)	O4B—C17B	1.3673 (17)
O4A—C20A	1.4329 (17)	O4B—C20B	1.4346 (17)
O5A—C16A	1.3733 (18)	O5B—C16B	1.3875 (16)
O5A—C21A	1.4247 (19)	O5B—C21B	1.434 (2)
C1A—C6A	1.340 (2)	C1B—C6B	1.346 (2)
C1A—C2A	1.4768 (19)	C1B—C2B	1.471 (2)
C1A—C13A	1.5102 (19)	C1B—C13B	1.511 (2)
C2A—C3A	1.512 (2)	C2B—C3B	1.512 (2)
C3A—C4A	1.532 (2)	C3B—C4B	1.537 (2)
C3A—H3AA	0.9900	C3B—H3BA	0.9900
C3A—H3AB	0.9900	C3B—H3BB	0.9900
C4A—C23A	1.528 (2)	C4B—C23B	1.527 (2)
C4A—C22A	1.537 (2)	C4B—C22B	1.535 (2)
C4A—C5A	1.5390 (19)	C4B—C5B	1.538 (2)
C5A—C6A	1.489 (2)	C5B—C6B	1.488 (2)
C5A—H5AA	0.9900	C5B—H5BA	0.9900
C5A—H5AB	0.9900	C5B—H5BB	0.9900
C7A—C12A	1.340 (2)	C7B—C12B	1.344 (2)
C7A—C8A	1.4983 (19)	C7B—C8B	1.494 (2)
C8A—C9A	1.540 (2)	C8B—C9B	1.530 (2)
C8A—H8AA	0.9900	C8B—H8BA	0.9900
C8A—H8AB	0.9900	C8B—H8BB	0.9900
C9A—C10A	1.528 (2)	C9B—C25B	1.528 (2)
C9A—C25A	1.534 (2)	C9B—C24B	1.535 (2)
C9A—C24A	1.535 (2)	C9B—C10B	1.538 (2)
C10A—C11A	1.515 (2)	C10B—C11B	1.511 (2)
C10A—H10A	0.9900	C10B—H10C	0.9900
C10A—H10B	0.9900	C10B—H10D	0.9900
C11A—C12A	1.4730 (19)	C11B—C12B	1.474 (2)
C12A—C13A	1.5167 (19)	C12B—C13B	1.511 (2)
C13A—C14A	1.5289 (19)	C13B—C14B	1.530 (2)
C13A—H13A	1.0000	C13B—H13B	1.0000
C14A—C19A	1.3813 (19)	C14B—C19B	1.391 (2)
C14A—C15A	1.399 (2)	C14B—C15B	1.4026 (19)
C15A—C16A	1.384 (2)	C15B—C16B	1.380 (2)
C15A—H15A	0.9500	C15B—H15B	0.9500
C16A—C17A	1.408 (2)	C16B—C17B	1.4090 (19)
C17A—C18A	1.383 (2)	C17B—C18B	1.394 (2)
C18A—C19A	1.396 (2)	C18B—C19B	1.393 (2)
C18A—H18A	0.9500	C18B—H18B	0.9500
C19A—H19A	0.9500	C19B—H19B	0.9500
C20A—H20A	0.9800	C20B—H20D	0.9800
C20A—H20B	0.9800	C20B—H20E	0.9800
C20A—H20C	0.9800	C20B—H20F	0.9800
C21A—H21A	0.9800	C21B—H21D	0.9800
C21A—H21B	0.9800	C21B—H21E	0.9800
C21A—H21C	0.9800	C21B—H21F	0.9800
C22A—H22A	0.9800	C22B—H22D	0.9800
C22A—H22B	0.9800	C22B—H22E	0.9800
C22A—H22C	0.9800	C22B—H22F	0.9800
C23A—H23A	0.9800	C23B—H23D	0.9800
C23A—H23B	0.9800	C23B—H23E	0.9800
C23A—H23C	0.9800	C23B—H23G	0.9800
C24A—H24A	0.9800	C24B—H24D	0.9800
C24A—H24B	0.9800	C24B—H24G	0.9800
C24A—H24C	0.9800	C24B—H24E	0.9800
C25A—H25A	0.9800	C25B—H25D	0.9800
C25A—H25B	0.9800	C25B—H25E	0.9800
C25A—H25C	0.9800	C25B—H25F	0.9800
			
C7A—O1A—C6A	117.69 (11)	C7B—O1B—C6B	117.46 (11)
C17A—O4A—C20A	116.72 (12)	C17B—O4B—C20B	116.61 (11)
C16A—O5A—C21A	116.35 (12)	C16B—O5B—C21B	112.54 (11)
C6A—C1A—C2A	118.52 (12)	C6B—C1B—C2B	118.46 (14)
C6A—C1A—C13A	122.70 (12)	C6B—C1B—C13B	122.33 (14)
C2A—C1A—C13A	118.71 (12)	C2B—C1B—C13B	119.20 (12)
O2A—C2A—C1A	120.48 (13)	O2B—C2B—C1B	120.67 (14)
O2A—C2A—C3A	121.12 (13)	O2B—C2B—C3B	121.21 (14)
C1A—C2A—C3A	118.36 (12)	C1B—C2B—C3B	118.04 (13)
C2A—C3A—C4A	114.67 (12)	C2B—C3B—C4B	115.09 (12)
C2A—C3A—H3AA	108.6	C2B—C3B—H3BA	108.5
C4A—C3A—H3AA	108.6	C4B—C3B—H3BA	108.5
C2A—C3A—H3AB	108.6	C2B—C3B—H3BB	108.5
C4A—C3A—H3AB	108.6	C4B—C3B—H3BB	108.5
H3AA—C3A—H3AB	107.6	H3BA—C3B—H3BB	107.5
C23A—C4A—C3A	109.81 (12)	C23B—C4B—C22B	109.54 (13)
C23A—C4A—C22A	109.05 (13)	C23B—C4B—C3B	109.74 (12)
C3A—C4A—C22A	110.56 (12)	C22B—C4B—C3B	110.21 (12)
C23A—C4A—C5A	108.77 (12)	C23B—C4B—C5B	108.79 (12)
C3A—C4A—C5A	108.24 (12)	C22B—C4B—C5B	110.42 (12)
C22A—C4A—C5A	110.40 (12)	C3B—C4B—C5B	108.10 (12)
C6A—C5A—C4A	112.43 (12)	C6B—C5B—C4B	112.54 (12)
C6A—C5A—H5AA	109.1	C6B—C5B—H5BA	109.1
C4A—C5A—H5AA	109.1	C4B—C5B—H5BA	109.1
C6A—C5A—H5AB	109.1	C6B—C5B—H5BB	109.1
C4A—C5A—H5AB	109.1	C4B—C5B—H5BB	109.1
H5AA—C5A—H5AB	107.9	H5BA—C5B—H5BB	107.8
C1A—C6A—O1A	123.02 (12)	C1B—C6B—O1B	122.38 (14)
C1A—C6A—C5A	125.53 (12)	C1B—C6B—C5B	126.03 (14)
O1A—C6A—C5A	111.45 (12)	O1B—C6B—C5B	111.58 (12)
C12A—C7A—O1A	123.02 (12)	C12B—C7B—O1B	122.74 (14)
C12A—C7A—C8A	125.29 (13)	C12B—C7B—C8B	125.82 (14)
O1A—C7A—C8A	111.68 (12)	O1B—C7B—C8B	111.44 (12)
C7A—C8A—C9A	111.83 (12)	C7B—C8B—C9B	112.29 (12)
C7A—C8A—H8AA	109.3	C7B—C8B—H8BA	109.1
C9A—C8A—H8AA	109.3	C9B—C8B—H8BA	109.1
C7A—C8A—H8AB	109.3	C7B—C8B—H8BB	109.1
C9A—C8A—H8AB	109.3	C9B—C8B—H8BB	109.1
H8AA—C8A—H8AB	107.9	H8BA—C8B—H8BB	107.9
C10A—C9A—C25A	109.77 (13)	C25B—C9B—C8B	109.18 (13)
C10A—C9A—C24A	110.97 (13)	C25B—C9B—C24B	109.33 (13)
C25A—C9A—C24A	108.99 (13)	C8B—C9B—C24B	110.65 (13)
C10A—C9A—C8A	107.73 (12)	C25B—C9B—C10B	110.53 (13)
C25A—C9A—C8A	109.27 (12)	C8B—C9B—C10B	107.64 (12)
C24A—C9A—C8A	110.09 (13)	C24B—C9B—C10B	109.49 (13)
C11A—C10A—C9A	115.36 (12)	C11B—C10B—C9B	112.29 (12)
C11A—C10A—H10A	108.4	C11B—C10B—H10C	109.1
C9A—C10A—H10A	108.4	C9B—C10B—H10C	109.1
C11A—C10A—H10B	108.4	C11B—C10B—H10D	109.1
C9A—C10A—H10B	108.4	C9B—C10B—H10D	109.1
H10A—C10A—H10B	107.5	H10C—C10B—H10D	107.9
O3A—C11A—C12A	120.24 (13)	O3B—C11B—C12B	120.67 (15)
O3A—C11A—C10A	121.64 (13)	O3B—C11B—C10B	122.39 (14)
C12A—C11A—C10A	118.08 (13)	C12B—C11B—C10B	116.91 (13)
C7A—C12A—C11A	118.74 (12)	C7B—C12B—C11B	118.63 (14)
C7A—C12A—C13A	122.70 (13)	C7B—C12B—C13B	122.00 (14)
C11A—C12A—C13A	118.55 (12)	C11B—C12B—C13B	119.35 (13)
C1A—C13A—C12A	108.84 (11)	C12B—C13B—C1B	108.13 (12)
C1A—C13A—C14A	109.54 (11)	C12B—C13B—C14B	110.72 (12)
C12A—C13A—C14A	111.20 (11)	C1B—C13B—C14B	112.55 (12)
C1A—C13A—H13A	109.1	C12B—C13B—H13B	108.4
C12A—C13A—H13A	109.1	C1B—C13B—H13B	108.4
C14A—C13A—H13A	109.1	C14B—C13B—H13B	108.4
C19A—C14A—C15A	119.31 (13)	C19B—C14B—C15B	117.90 (13)
C19A—C14A—C13A	121.78 (13)	C19B—C14B—C13B	121.54 (13)
C15A—C14A—C13A	118.89 (12)	C15B—C14B—C13B	120.51 (12)
C16A—C15A—C14A	120.59 (13)	C16B—C15B—C14B	121.26 (13)
C16A—C15A—H15A	119.7	C16B—C15B—H15B	119.4
C14A—C15A—H15A	119.7	C14B—C15B—H15B	119.4
O5A—C16A—C15A	124.95 (13)	C15B—C16B—O5B	119.43 (13)
O5A—C16A—C17A	115.49 (13)	C15B—C16B—C17B	120.43 (13)
C15A—C16A—C17A	119.56 (14)	O5B—C16B—C17B	120.12 (13)
O4A—C17A—C18A	124.90 (13)	O4B—C17B—C18B	125.18 (13)
O4A—C17A—C16A	115.18 (13)	O4B—C17B—C16B	116.13 (12)
C18A—C17A—C16A	119.91 (13)	C18B—C17B—C16B	118.69 (13)
C17A—C18A—C19A	119.82 (13)	C19B—C18B—C17B	120.14 (13)
C17A—C18A—H18A	120.1	C19B—C18B—H18B	119.9
C19A—C18A—H18A	120.1	C17B—C18B—H18B	119.9
C14A—C19A—C18A	120.80 (14)	C14B—C19B—C18B	121.58 (13)
C14A—C19A—H19A	119.6	C14B—C19B—H19B	119.2
C18A—C19A—H19A	119.6	C18B—C19B—H19B	119.2
O4A—C20A—H20A	109.5	O4B—C20B—H20D	109.5
O4A—C20A—H20B	109.5	O4B—C20B—H20E	109.5
H20A—C20A—H20B	109.5	H20D—C20B—H20E	109.5
O4A—C20A—H20C	109.5	O4B—C20B—H20F	109.5
H20A—C20A—H20C	109.5	H20D—C20B—H20F	109.5
H20B—C20A—H20C	109.5	H20E—C20B—H20F	109.5
O5A—C21A—H21A	109.5	O5B—C21B—H21D	109.5
O5A—C21A—H21B	109.5	O5B—C21B—H21E	109.5
H21A—C21A—H21B	109.5	H21D—C21B—H21E	109.5
O5A—C21A—H21C	109.5	O5B—C21B—H21F	109.5
H21A—C21A—H21C	109.5	H21D—C21B—H21F	109.5
H21B—C21A—H21C	109.5	H21E—C21B—H21F	109.5
C4A—C22A—H22A	109.5	C4B—C22B—H22D	109.5
C4A—C22A—H22B	109.5	C4B—C22B—H22E	109.5
H22A—C22A—H22B	109.5	H22D—C22B—H22E	109.5
C4A—C22A—H22C	109.5	C4B—C22B—H22F	109.5
H22A—C22A—H22C	109.5	H22D—C22B—H22F	109.5
H22B—C22A—H22C	109.5	H22E—C22B—H22F	109.5
C4A—C23A—H23A	109.5	C4B—C23B—H23D	109.5
C4A—C23A—H23B	109.5	C4B—C23B—H23E	109.5
H23A—C23A—H23B	109.5	H23D—C23B—H23E	109.5
C4A—C23A—H23C	109.5	C4B—C23B—H23G	109.5
H23A—C23A—H23C	109.5	H23D—C23B—H23G	109.5
H23B—C23A—H23C	109.5	H23E—C23B—H23G	109.5
C9A—C24A—H24A	109.5	C9B—C24B—H24D	109.5
C9A—C24A—H24B	109.5	C9B—C24B—H24G	109.5
H24A—C24A—H24B	109.5	H24D—C24B—H24G	109.5
C9A—C24A—H24C	109.5	C9B—C24B—H24E	109.5
H24A—C24A—H24C	109.5	H24D—C24B—H24E	109.5
H24B—C24A—H24C	109.5	H24G—C24B—H24E	109.5
C9A—C25A—H25A	109.5	C9B—C25B—H25D	109.5
C9A—C25A—H25B	109.5	C9B—C25B—H25E	109.5
H25A—C25A—H25B	109.5	H25D—C25B—H25E	109.5
C9A—C25A—H25C	109.5	C9B—C25B—H25F	109.5
H25A—C25A—H25C	109.5	H25D—C25B—H25F	109.5
H25B—C25A—H25C	109.5	H25E—C25B—H25F	109.5
			
C6A—C1A—C2A—O2A	174.17 (14)	C6B—C1B—C2B—O2B	175.45 (14)
C13A—C1A—C2A—O2A	−8.7 (2)	C13B—C1B—C2B—O2B	−3.2 (2)
C6A—C1A—C2A—C3A	−3.5 (2)	C6B—C1B—C2B—C3B	−1.2 (2)
C13A—C1A—C2A—C3A	173.59 (13)	C13B—C1B—C2B—C3B	−179.85 (13)
O2A—C2A—C3A—C4A	156.42 (14)	O2B—C2B—C3B—C4B	155.50 (14)
C1A—C2A—C3A—C4A	−25.91 (19)	C1B—C2B—C3B—C4B	−27.87 (19)
C2A—C3A—C4A—C23A	169.58 (12)	C2B—C3B—C4B—C23B	169.74 (13)
C2A—C3A—C4A—C22A	−70.06 (16)	C2B—C3B—C4B—C22B	−69.55 (17)
C2A—C3A—C4A—C5A	50.97 (16)	C2B—C3B—C4B—C5B	51.22 (17)
C23A—C4A—C5A—C6A	−168.10 (13)	C23B—C4B—C5B—C6B	−166.56 (12)
C3A—C4A—C5A—C6A	−48.84 (16)	C22B—C4B—C5B—C6B	73.19 (16)
C22A—C4A—C5A—C6A	72.29 (16)	C3B—C4B—C5B—C6B	−47.44 (16)
C2A—C1A—C6A—O1A	−175.93 (13)	C2B—C1B—C6B—O1B	−175.04 (13)
C13A—C1A—C6A—O1A	7.1 (2)	C13B—C1B—C6B—O1B	3.6 (2)
C2A—C1A—C6A—C5A	4.7 (2)	C2B—C1B—C6B—C5B	3.8 (2)
C13A—C1A—C6A—C5A	−172.24 (13)	C13B—C1B—C6B—C5B	−177.60 (14)
C7A—O1A—C6A—C1A	5.0 (2)	C7B—O1B—C6B—C1B	13.7 (2)
C7A—O1A—C6A—C5A	−175.62 (12)	C7B—O1B—C6B—C5B	−165.29 (12)
C4A—C5A—C6A—C1A	23.1 (2)	C4B—C5B—C6B—C1B	22.4 (2)
C4A—C5A—C6A—O1A	−156.26 (12)	C4B—C5B—C6B—O1B	−158.66 (12)
C6A—O1A—C7A—C12A	−6.9 (2)	C6B—O1B—C7B—C12B	−11.9 (2)
C6A—O1A—C7A—C8A	172.65 (12)	C6B—O1B—C7B—C8B	167.92 (12)
C12A—C7A—C8A—C9A	−27.0 (2)	C12B—C7B—C8B—C9B	−17.6 (2)
O1A—C7A—C8A—C9A	153.46 (12)	O1B—C7B—C8B—C9B	162.62 (12)
C7A—C8A—C9A—C10A	50.47 (16)	C7B—C8B—C9B—C25B	167.61 (12)
C7A—C8A—C9A—C25A	169.68 (13)	C7B—C8B—C9B—C24B	−72.01 (16)
C7A—C8A—C9A—C24A	−70.65 (16)	C7B—C8B—C9B—C10B	47.59 (16)
C25A—C9A—C10A—C11A	−169.89 (13)	C25B—C9B—C10B—C11B	−177.59 (13)
C24A—C9A—C10A—C11A	69.58 (16)	C8B—C9B—C10B—C11B	−58.43 (16)
C8A—C9A—C10A—C11A	−51.00 (16)	C24B—C9B—C10B—C11B	61.91 (17)
C9A—C10A—C11A—O3A	−156.99 (14)	C9B—C10B—C11B—O3B	−144.04 (15)
C9A—C10A—C11A—C12A	25.40 (19)	C9B—C10B—C11B—C12B	38.10 (18)
O1A—C7A—C12A—C11A	178.17 (13)	O1B—C7B—C12B—C11B	174.40 (12)
C8A—C7A—C12A—C11A	−1.3 (2)	C8B—C7B—C12B—C11B	−5.4 (2)
O1A—C7A—C12A—C13A	−3.2 (2)	O1B—C7B—C12B—C13B	−7.2 (2)
C8A—C7A—C12A—C13A	177.30 (14)	C8B—C7B—C12B—C13B	173.05 (13)
O3A—C11A—C12A—C7A	−175.04 (14)	O3B—C11B—C12B—C7B	176.85 (14)
C10A—C11A—C12A—C7A	2.6 (2)	C10B—C11B—C12B—C7B	−5.3 (2)
O3A—C11A—C12A—C13A	6.3 (2)	O3B—C11B—C12B—C13B	−1.6 (2)
C10A—C11A—C12A—C13A	−176.08 (13)	C10B—C11B—C12B—C13B	176.29 (12)
C6A—C1A—C13A—C12A	−15.15 (19)	C7B—C12B—C13B—C1B	21.57 (18)
C2A—C1A—C13A—C12A	167.88 (12)	C11B—C12B—C13B—C1B	−160.03 (12)
C6A—C1A—C13A—C14A	106.62 (15)	C7B—C12B—C13B—C14B	−102.17 (16)
C2A—C1A—C13A—C14A	−70.35 (16)	C11B—C12B—C13B—C14B	76.23 (16)
C7A—C12A—C13A—C1A	13.27 (19)	C6B—C1B—C13B—C12B	−19.82 (19)
C11A—C12A—C13A—C1A	−168.10 (12)	C2B—C1B—C13B—C12B	158.77 (12)
C7A—C12A—C13A—C14A	−107.48 (16)	C6B—C1B—C13B—C14B	102.80 (16)
C11A—C12A—C13A—C14A	71.14 (16)	C2B—C1B—C13B—C14B	−78.61 (16)
C1A—C13A—C14A—C19A	120.01 (14)	C12B—C13B—C14B—C19B	−123.15 (15)
C12A—C13A—C14A—C19A	−119.65 (14)	C1B—C13B—C14B—C19B	115.69 (15)
C1A—C13A—C14A—C15A	−58.45 (16)	C12B—C13B—C14B—C15B	54.28 (18)
C12A—C13A—C14A—C15A	61.90 (16)	C1B—C13B—C14B—C15B	−66.88 (18)
C19A—C14A—C15A—C16A	−0.4 (2)	C19B—C14B—C15B—C16B	0.4 (2)
C13A—C14A—C15A—C16A	178.06 (12)	C13B—C14B—C15B—C16B	−177.13 (14)
C21A—O5A—C16A—C15A	4.6 (2)	C14B—C15B—C16B—O5B	−178.82 (14)
C21A—O5A—C16A—C17A	−175.41 (14)	C14B—C15B—C16B—C17B	−0.2 (2)
C14A—C15A—C16A—O5A	−179.48 (13)	C21B—O5B—C16B—C15B	−103.40 (16)
C14A—C15A—C16A—C17A	0.5 (2)	C21B—O5B—C16B—C17B	78.01 (17)
C20A—O4A—C17A—C18A	−1.9 (2)	C20B—O4B—C17B—C18B	−6.1 (2)
C20A—O4A—C17A—C16A	177.96 (12)	C20B—O4B—C17B—C16B	173.68 (13)
O5A—C16A—C17A—O4A	−0.01 (18)	C15B—C16B—C17B—O4B	−179.75 (14)
C15A—C16A—C17A—O4A	179.99 (12)	O5B—C16B—C17B—O4B	−1.2 (2)
O5A—C16A—C17A—C18A	179.82 (13)	C15B—C16B—C17B—C18B	0.1 (2)
C15A—C16A—C17A—C18A	−0.2 (2)	O5B—C16B—C17B—C18B	178.66 (13)
O4A—C17A—C18A—C19A	179.58 (13)	O4B—C17B—C18B—C19B	179.72 (14)
C16A—C17A—C18A—C19A	−0.2 (2)	C16B—C17B—C18B—C19B	−0.1 (2)
C15A—C14A—C19A—C18A	0.0 (2)	C15B—C14B—C19B—C18B	−0.4 (2)
C13A—C14A—C19A—C18A	−178.43 (12)	C13B—C14B—C19B—C18B	177.09 (14)
C17A—C18A—C19A—C14A	0.3 (2)	C17B—C18B—C19B—C14B	0.3 (2)

### Hydrogen-bond geometry (Å, °)

**Table:**

*D*—H···*A*	*D*—H	H···*A*	*D*···*A*	*D*—H···*A*
C10A—H10A···O4A^i^	0.99	2.38	3.2817 (19)	152
C18A—H18A···O2A^ii^	0.95	2.35	3.2943 (19)	176
C18B—H18B···O2B^iii^	0.95	2.45	3.4000 (19)	175
C20A—H20A···O3A^iv^	0.98	2.59	3.4144 (19)	142
C24B—H24D···O4A^v^	0.98	2.48	3.453 (2)	171

Symmetry codes: (i) *x*, *y*−1, *z*; (ii) −*x*, −*y*+1, −*z*; (iii) −*x*+1, −*y*, −*z*+1; (iv) *x*, *y*+1, *z*; (v) −*x*, −*y*+1, −*z*+1.
